# Parallel assessment of albuminuria and plasma sTNFR1 in people with type 2 diabetes and advanced chronic kidney disease provides accurate prognostication of the risks of renal decline and death

**DOI:** 10.1038/s41598-020-71684-6

**Published:** 2020-09-09

**Authors:** William P. Martin, Colm Tuohy, Alison Doody, Sabrina Jackson, Ronan J. Canavan, David Slattery, Patrick J. Twomey, Malachi J. McKenna, Carel W. le Roux, Neil G. Docherty

**Affiliations:** 1 Diabetes Complications Research Centre, School of Medicine, Conway Institute of Biomolecular and Biomedical Research, University College Dublin, Belfield, Dublin 4, Ireland; 2 Department of Endocrinology, St. Vincent’s University Hospital, Elm Park, Dublin 4, Ireland; 3 Department of Clinical Chemistry, St. Vincent’s University Hospital, Elm Park, Dublin 4, Ireland; 4 Institute of Clinical Sciences, Sahlgrenska Academy, University of Gothenburg, Gothenburg, Sweden; 5 Division of Investigative Science, Imperial College London, London, UK

**Keywords:** Chronic kidney disease, Diabetes complications, Prognostic markers

## Abstract

Identification of people with diabetes and chronic kidney disease at high-risk of early mortality is a priority to guide intensification of therapy. We aimed to investigate the complementary prognostic value of baseline urine albumin-to-creatinine ratio (uACR) and plasma soluble tumour necrosis factor receptor-1 (sTNFR1) with respect to early mortality and renal functional decline in a population with type 2 diabetes and advanced chronic kidney disease. We measured plasma sTNFR1 in people with type 2 diabetes (HbA_1c_ ≥ 48 mmol/mol) at 2 hospital sites in Dublin between October 15th, 2014 and July 17th, 2015. In a subgroup of patients with advanced chronic kidney disease at baseline (estimated glomerular filtration rate (eGFR) ≤ 60 mL/min/BSA) (n = 118), we collected clinical and longitudinal laboratory data to investigate relationships between sTNFR1 and renal and mortality endpoints by multivariable linear mixed-effects models and Cox proportional hazards regression models. The cohort was 64% male and 97% Caucasian. Mean age was 74 years, with a median type 2 diabetes duration of 16 years. Mean CKD-EPI eGFR was 42 mL/min/BSA and median [IQR] uACR was 3 [11] mg/mmol. Twenty-three (39%) people in quartiles 3 and 4 for plasma sTNFR1 died over 4-year follow-up. After adjustment for clinical variables, annual CKD-EPI eGFR decreased by − 0.56 mL/min/BSA/year for each logarithm unit increase in baseline uACR, corresponding to an annual loss of renal function of 3% per year. Furthermore, elevated uACR, but not sTNFR1, increased the risk of ≥ 40% decline in CKD-EPI eGFR (HR 1.5, p = 0.001) and doubling of serum creatinine (HR 2.0, p < 0.001). Plasma sTNFR1 did not predict a more negative trajectory in eGFR slope. However, for those people in quartiles 3 and 4 for plasma sTNFR1, an increased risk of incident mortality was detected (HR 4.9, p = 0.02). No such association was detected for uACR. In this elderly cohort of patients with type 2 diabetes and chronic kidney disease, sTNFR1 predicted short-to-medium term mortality risk but not risk of progressive renal functional decline. In contrast, parallel assessment of uACR predicted renal functional decline but not mortality, highlighting the complementary prognostic information provided by both parameters.

## Background

Diabetes is a major modifiable risk factor for cardiovascular disease and all-cause mortality, with the majority of excess mortality attributable to diabetes occurring in those with kidney disease^1^. Diabetes was the primary cause of end-stage kidney disease (ESKD) in 38.6% of the United States ESKD population in 2017^2^. Over the past decade, new type 2 diabetes therapies including glucagon-like peptide-1 receptor analogues (GLP1RAs) and sodium-glucose co-transporter-2 inhibitors (SGLT2is) have been demonstrated to exert both cardioprotective and renoprotective effects^3,4^. Enhanced integration of diabetes and nephrology care, including earlier referral to nephrology services, can improve renal outcomes for people with diabetes and chronic kidney disease (CKD)^5^. Intentional weight loss strategies, including metabolic surgery, may also reduce the burden of cardiovascular disease and slow renal functional decline in people with type 2 diabetes and CKD^6–8^. Given the range of new and efficacious options for the treatment of diabetes and its complications, identifying subgroups at the highest risk of rapid renal functional decline and/or death will help clinicians to appropriately stratify intensification of therapy.

Plasma soluble tumour necrosis factor receptor-1 (sTNFR1/CD120a) is a circulating protein reflective of systemic inflammation and which is increased in diabetic kidney disease (DKD)^9–11^. Plasma sTNFR1 positively correlates with podocyte foot process effacement and predicts accelerated renal functional decline, ESKD, and increased cardiovascular and all-cause mortality risk in people with diabetes^12–18^. The extent to which the prognostic value of plasma sTNFR1 for renal and mortality outcomes extends to elderly people with type 2 diabetes and well advanced CKD remains to be defined. In the present study, we investigated the independent relationships between sTNFR1 and both all-cause mortality and accelerated renal functional decline and ESKD. We report on both individual and composite endpoints in this regard, in a population enriched for such events (elderly population with longstanding type 2 diabetes and CKD stage 3 or greater).

## Methods

### Study cohort

Permission was obtained from St Vincent’s Healthcare Group, Dublin, Ireland for two subsequent prospective clinical audits (reference numbers 2014/1103 and 2019/2324) evaluating reporting practice on biochemical risk factors for microvascular and macrovascular disease in people with diabetes (HbA_1c_, lipid profile, eGFR, and albuminuria). Contemporaneous to establishment of the audit we instigated measurement of sTNFR1 as part of routine care on clinical grounds in people with diabetes during 2014 and 2015 (suboptimal glycaemic control as defined by HbA_1c_ ≥ 48 mmol/mol). We previously reported cross-sectional associations between plasma sTNFR1 and renal injury in this cohort^19^. Herein we report on the ability of plasma sTNFR1 to predict mortality and renal endpoints at follow-up in a subgroup of 118 people with established type 2 diabetes and CKD (baseline Chronic Kidney Disease-Epidemiology Collaboration (CKD-EPI) eGFR ≤ 60 mL/min/BSA). Individuals with type 1 diabetes or active malignancy (save for non-melanoma skin cancers) at study enrolment were excluded. The performance of sTNFR1 as a predictor of both mortality and renal end-points was compared and contrasted to that of the routine clinical measure of urinary albumin-to-creatinine ratio (uACR). People with ESKD on renal replacement therapy or with a prior kidney transplant were excluded.

### Clinical information

Plasma sTNFR1 was routinely measured between October 15th, 2014 and July 17th, 2015. Longitudinal clinical and laboratory data were obtained from October 15th, 2014 to the end of follow-up on August 31st, 2019. Clinical information (demographics, anthropometry, smoking status, type 2 diabetes duration, blood pressure, diabetes complications, comorbidities, and medication usage) was extracted for each participant from an electronic health record for people with diabetes (TYMAX), as well as from hospital discharge and outpatient records entered into medical charts. Clinical indices were assigned to a specific date. If the exact date of commencement/onset was not available (eg, prescription of non-diabetes medications), the indices were recorded as being either present or absent before the end of longitudinal follow-up on August 31st, 2019.

### Biochemical measures

As previously described, sTNFR1 was measured in tenfold diluted plasma samples using the EKF Diagnostics Human sTNFR1 ELISA assay (Cat# EIA—BIO94; EKF Diagnostics Ltd., Cardiff, UK)^19^. Assay run validity was established based on pass/fail criteria for technical duplicates for low (290 pg/ml) and high (2834 pg/ml) control test concentrations resulting in an overall inter-assay coefficient of variation of 6%^19^.

All recorded clinical biochemistry tests were analysed via routine clinical biochemistry services at St Vincent’s University Hospital, Dublin. Glycated hemoglobin (HbA_1c_) was measured by high-performance liquid chromatography (A. Menarini Diagnostics HA-8180 V). Creatinine and albumin were measured on a Roche/Hitachi Cobas c502 modular auto-analyser. The creatinine assay changed from the Jaffe method to an enzymatic assay in January 2016 (both assays were IDMS traceable). Longitudinal laboratory data was manually abstracted from the hospital laboratory information systems.

CKD-EPI eGFR was calculated from serum creatinine using standard formulae and expressed as mL/min/body surface area (BSA)^20,21^. Only patients with ≥ 3 eGFR determinations over ≥ 1 year were included in analyses of longitudinal renal functional data. Sensitivity analyses were also performed in which only individuals with ≥ 3 eGFR determinations over ≥ 2 years were included in regression models of renal outcomes. Serum creatinine values subsequent to renal replacement therapy initiation were excluded. Annual slopes of CKD-EPI eGFR were calculated by linear regression of eGFR over time in years. Determination of mortality, renal (≥ 40% decline in CKD-EPI eGFR, doubling of serum creatinine, need for renal replacement therapy), and composite endpoints was performed manually for each patient on a case-by-case basis. Cause of death was categorised as cardiovascular, neoplastic, or infectious based on the primary aetiology. Time to event was recorded for each relevant endpoint reached.

### Statistical analyses

RStudio version 3.6.1 was used for analysis. Baseline characteristics at study enrolment and clinical characteristics of the cohort during follow-up (renal and mortality endpoints) were summarised by descriptive statistics. Categorical variables are presented as frequencies and percentages, and were compared across plasma sTNFR1 quartiles using χ^2^ tests. Continuous variables with normal and skewed distributions are presented as mean ± SD and median [interquartile range], respectively. One-way between-groups ANOVAs and Kruskal–Wallis tests were used to assess for differences across plasma sTNFR1 quartiles in continuous variables with normal and skewed distributions, respectively. P < 0.05 was considered statistically significant.

Diabetes duration, urine albumin-to-creatinine ratio (uACR), and plasma sTNFR1 were log-transformed for regression analyses due to non-Gaussian distributions. Univariate relationships between uACR and sTNFR1 with ≥ 40% decline in CKD-EPI eGFR, doubling of serum creatinine, and mortality were investigated using logistic regression. Multivariable Cox proportional hazards and logistic regression models were created to further investigate relationships between uACR, and sTNFR1 with renal endpoints and mortality. Two models were constructed for each endpoint: firstly, a clinical model adjusting for conventional risk factors for renal functional decline including age, gender, diabetes duration, systolic blood pressure, HbA_1c_, CKD-EPI eGFR, and uACR; and secondly a clinical + sTNFR1 model incorporating the aforementioned variables and plasma sTNFR1. Cox models were constructed using the R package survival^22^; unadjusted (Kaplan–Meier) and adjusted survival curves were plotted using the R package survminer according to sTNFR1 quartiles^23^. Comparisons between sTNFR1 quartiles on the Kaplan–Meier plot were made using the log-rank test. We tested each Cox proportional hazards model for proportionality assumptions using Schoenfeld residuals.

Cox model results are presented as the hazard ratio (HR), 95% confidence interval (CI), and p-value. Logistic regression model results are presented as the odds ratio (OR), 95% CI, and p-value. Comparisons of model adequacy (clinical model versus clinical + sTNFR1 model) were assessed for Cox and logistic regression models using likelihood ratio χ^2^ tests. We used the competing risk model of Fine and Gray to estimate the subdistribution HRs for ≥ 40% decline in CKD-EPI eGFR and doubling of serum creatinine while accounting for the competing risk of all-cause mortality. The function crr from the R package cmprsk was used to construct Fine-Gray models^24^. Plots of Schoenfeld residuals versus time failure for each term in the models were used to examine the proportional hazard sub-distribution assumption. Cumulative incidence of events of interest (≥ 40% decline in CKD-EPI eGFR and doubling of serum creatinine) and the competing risk of death were calculated using the cuminc function in the R package cmprsk^24^. Cumulative incidence plots were generated using ggcompetingrisks from the R package survminer^23^.

Linear mixed-effects models were constructed to investigate relationships between uACR and sTNFR1 with annual changes in CKD-EPI eGFR. Separate models were fitted for CKD-EPI eGFR and log-transformed CKD-EPI eGFR. Similarly, a clinical model was constructed that adjusted for the same clinical fixed effects as the aforementioned Cox proportional hazards and logistic regression models, and a clinical + sTNFR1 model which was additionally adjusted for plasma sTNFR1. All linear mixed-effects models incorporated subject-specific random intercepts and slopes for the duration of renal functional follow-up. Models fitted to the absolute eGFR values determine yearly changes in renal function in native units (mL/min/BSA/year), whereas models fitted using log-transformed eGFR estimate annual percentage changes in renal function. Comparisons of model adequacy (clinical model versus clinical + sTNFR1 model) were assessed for linear mixed-effects models using likelihood ratio χ^2^ tests. The function lmer (from the R package lmerTest) was used to fit and test the models^25^. No serious violations of logistic regression or linear mixed-effects models was found on examination of the distribution of residuals.

### Ethics approval and consent to participate

The study was approved by the clinical audit committee at St. Vincent’s Healthcare Group, Dublin, Ireland (reference numbers 2014/1103 and 2019/2324). Under clinical audit guidelines, informed consent from individual participants was not obtained but all data was handled as per General Data Protection Regulation guidelines (EU), 2016/679. The need for informed consent was waived by the St. Vincent’s Healthcare Group research ethics committee. All procedures performed were in accordance with the ethical standards of the institutional audit committee and with the 1964 Helsinki declaration and its later amendments.

## Results

### Baseline characteristics

Table 1 presents baseline characteristics of the study cohort (n = 118) according to sTNFR1 quartile (n = 29–30 per quartile). The study population had a mean age of 74 years, and predominantly consisted of Caucasian subjects with longstanding type 2 diabetes. The study population had advanced CKD at baseline (mean CKD-EPI eGFR 41.7 ± 11.4 mL/min/BSA), with baseline renal function steadily declining from quartile 1 to quartile 4 of sTNFR1. Baseline albuminuria was higher and glycaemic control worsened in those people in quartile 4 for plasma sTNFR1.

**Table 1:** Baseline characteristics of the study cohort stratified by plasma sTNFR1 quartiles (n = 118)^a,^^b^.

Characteristic	Data available (n (%))	Total cohort (n = 118)	sTNFR1 Q1 (n = 30)	sTNFR1 Q2 (n = 29)	sTNFR1 Q3 (n = 30)	sTNFR1 Q4 (n = 29)	p
Plasma sTNFR1 (min–max; pg/mL)	118 (100)	553–10,606	553–1,941	1,956–2,635	2,654–3,555	3,646–10,606	N/A
Plasma sTNFR1 (median [IQR]; pg/mL)	118 (100)	2,644.5 [1604.0]	1536.5 [528.0]	2,324.0 [460.0]	3,088.0 [431.8]	4,562.0 [1194.0]	** < 0.001**
**Demographics**							
Age (mean ± SD; years)	118 (100)	73.9 ± 8.9	73.5 ± 10.3	75.0 ± 8.2	71.8 ± 7.7	75.3 ± 9.0	0.40^c^
Male (n (%))	118 (100)	75 (63.6)	18 (60.0)	22 (75.9)	16 (53.3)	19 (65.5)	0.33^d^
Caucasian ethnicity (n (%))	118 (100)	114 (96.6)	29 (96.7)	27 (93.1)	29 (96.7)	29 (100)	N/A^e^
Type 2 diabetes (n (%))	118 (100)	118 (100)	30 (100)	29 (100)	30 (100)	29 (100)	N/A
Diabetes duration (median [IQR]; years)	118 (100)	15.9 [10.8]	14.4 [10.1]	15.8 [11.6]	15.7 [11.9]	17.2 [9.5]	0.42^f^
Blood pressure (mean ± SD; mmHg)	118 (100)						
Systolic		135.9 ± 17.8	135.7 ± 20.8	134.5 ± 13.6	137.1 ± 16.2	136.1 ± 20.2	0.96
Diastolic		73.5 ± 10.6	74.4 ± 10.6	74.2 ± 8.9	73.3 ± 11.2	72.0 ± 11.9	0.82
Body-mass index (mean ± SD; kg/m^2^)	118 (100)	31.3 ± 9.5	29.2 ± 5.3	29.6 ± 4.6	35.4 ± 15.2	31.0 ± 7.6	**0.04**
Current smoker (n (%))	118 (100)	10 (8.5)	1 (3.3)	1 (3.5)	3 (10.0)	5 (17.2)	N/A
**Diabetes complications (n (%))**							
Coronary artery disease	115 (97.5)	21 (18.3)	2 (6.7)	6 (21.4)	7 (24.1)	6 (21.4)	0.29
Cerebrovascular disease	114 (96.6)	11 (9.7)	2 (6.7)	2 (7.1)	5 (17.2)	2 (7.4)	N/A
Peripheral arterial disease	116 (98.3)	21 (18.1)	4 (13.3)	4 (14.3)	8 (26.7)	5 (17.9)	0.53
Diabetic retinopathy	114 (96.6)	57 (50.0)	16 (55.2)	11 (37.9)	14 (48.3)	16 (59.3)	0.40
Diabetic neuropathy	115 (97.5)	34 (29.6)	10 (33.3)	10 (35.7)	7 (24.1)	7 (25.0)	0.70
**Laboratory results**							
Serum creatinine (mean ± SD; μmol/L)	118 (100)	141.9 ± 53.3	120.8 ± 26.2	128.3 ± 23.1	136.9 ± 38.8	182.4 ± 81.6	** < 0.001**
CKD-EPI eGFR (mean ± SD; mL/min/BSA)	118 (100)	41.7 ± 11.4	47.4 ± 8.7	45.3 ± 8.7	41.8 ± 10.4	32.2 ± 11.4	** < 0.001**
uACR (median [IQR]; mg/mmol)	101 (85.6)	3.0 [10.9]	2.9 [5.0]	1.9 [4.4]	2.1 [14.3]	35.5 [124.3]	**0.01**
HbA_1c_ (mean ± SD; mmol/mol)	118 (100)	59.8 ± 10.9	56.9 ± 8.6	56.9 ± 9.0	60.9 ± 11.2	64.6 ± 12.9	**0.02**
Haemoglobin (mean ± SD; g/dL)	117 (99.2)	12.7 ± 1.8	12.8 ± 1.6	13.4 ± 1.8	12.8 ± 1.5	11.8 ± 1.9	**0.01**
**Medications**	118 (100)						
GLP1RA (n (%))		13 (11.0)	3 (10.0)	3 (10.3)	5 (16.7)	2 (6.9)	N/A
SGLT2i (n (%))		2 (1.7)	0 (0)	2 (6.9)	0 (0)	0 (0)	N/A
Insulin (n (%))		50 (42.4)	9 (30.0)	9 (31.0)	15 (50.0)	17 (58.6)	**0.06**
Number of glucose-lowering medications (mean ± SD)		2.4 ± 1.2	2.5 ± 1.2	2.1 ± 1.3	2.4 ± 1.1	2.4 ± 1.0	0.58
Either ACE-inhibitor or ARB (n (%))		86 (72.9)	22 (73.3)	20 (69.0)	22 (73.3)	22 (75.9)	0.95
Number of antihypertensives (mean ± SD)		2.2 ± 1.1	2.0 ± 1.0	2.1 ± 1.3	2.2 ± 1.1	2.4 ± 1.1	0.62
Statin (n (%))		92 (78.0)	26 (86.7)	23 (79.3)	22 (73.3)	21 (72.4)	0.52
ESA (n (%))		0 (0)	0 (0)	0 (0)	0 (0)	0 (0)	N/A

^a^ACE, angiotensin-converting enzyme; ARB, angiotensin-II receptor blocker; CKD-EPI, Chronic Kidney Disease-Epidemiology Collaboration; eGFR, estimated glomerular filtration rate; ESA, erythropoietin-stimulating agent; GLP1RA, glucagon-like peptide-1 receptor analogue; HbA_1c_, glycated haemoglobin; IQR, interquartile range; N/A, not applicable; Q1, quartile 1; Q2, quartile 2; Q3, quartile 3; Q4, quartile 4; SD, standard deviation; SGLT2i, sodium-glucose co-transporter-2 inhibitor; sTNFR1, soluble tumour necrosis factor receptor-1; uACR, urine albumin-to-creatinine ratio.

^b^Values are given as n (%) for categorical variables, or mean ± SD for normally distributed continuous variables, unless otherwise indicated. Median [IQR] values are presented for continuous variables that are not normally distributed.

^c^One-way between-groups ANOVA was used to assess for variation in normally distributed continuous variables across the sTNFR1 quartiles.

^d^χ^2^ analysis was used to analyse for differences in categorical variables across the sTNFR1 quartiles.

^e^N/A indicates that the minimum expected cell frequency count for χ^2^ was not satisfied.

^f^Kruskall-Wallis test was used to assess for variation across the sTNFR1 quartiles in continuous variables that were not normally distributed.

Over 15% of the study population had coronary artery disease, while 50% had diabetic retinopathy. There were no significant differences in the presence of macrovascular or non-renal microvascular complications of diabetes across the sTNFR1 quartiles. Over 70% of the study cohort were treated with either an angiotensin-converting enzyme-inhibitor or angiotensin-II receptor blocker, and most people were on at least 2 antihypertensives. Usage of GLP1RAs and SGLT2is was low across all quartiles.

### Incidence of renal and mortality endpoints

Mean duration of renal functional follow-up was 3.5 ± 1.2 years, with each participant having a median of 14 eGFR determinations (Table 2). The median rate of annual decline in CKD-EPI eGFR was − 2.0 mL/min/BSA/year for the study cohort. Decline of CKD-EPI eGFR ≥ 40% trended to occur more frequently in people in sTNFR1 quartile 4 during follow-up, although this did not reach statistical significance (p = 0.06). Twenty-eight people (23.7% of the study cohort) died during follow-up. There was a linear increase in mortality rates from quartile 1 to 4 during follow-up (p < 0.001). Causes of death in our study cohort included n = 13 (46.4%) due to cardiovascular disease, n = 6 (21.4%) due to cancer, n = 5 (17.9%) due to infection, with cause of death missing in n = 4 (14.3%) cases. Over 50% of people in sTNFR1 quartile 4 died during follow-up. Consequently, the incidence of composite renal and mortality endpoints increased from quartile 1 to quartile 4 during follow-up, although this was mostly driven by high mortality rates in sTNFR1 quartiles 3–4 rather than a higher rate of interim renal endpoints.

**Table 2:** Duration of renal functional follow-up and incidence of renal and mortality endpoints during the study period (n = 118)^a,^^b^.

Characteristic	Data available (n (%))	Total cohort (n = 118)	sTNFR1 Q1 (n = 30)	sTNFR1 Q2 (n = 29)	sTNFR1 Q3 (n = 30)	sTNFR1 Q4 (n = 29)	p
**Duration of renal functional follow-up**	118 (100)						
Total number of eGFR measurements (median [IQR])		14.0 [16.0]	9.5 [17.0]	13.0 [14.0]	16.5 [16.3]	19.0 [16.0]	0.44^c^
Total duration of renal functional follow-up (mean ± SD; years)		3.5 ± 1.2	4.0 ± 0.7	3.8 ± 0.8	3.7 ± 1.1	2.5 ± 1.6	** < 0.001**^d^
**Renal endpoints**							
Slope of CKD-EPI eGFR (median [IQR]; ml/min/BSA/year)	109 (92.4)^e^	− 2.0 [3.1]	− 1.6 [4.0]	− 2.7 [2.2]	− 1.4 [2.9]	− 2.9 [3.9]	0.21
≥ 40% decrease in CKD-EPI eGFR during follow-up (n (%))	111 (94.1)^f^	31 (27.9)	8 (26.7)	7 (24.1)	5 (16.7)	11 (50.0)	0.06^ g^
Doubling of serum creatinine during follow-up (n (%))	111 (94.1)^f^	14 (12.6)	4 (13.3)	3 (10.3)	1 (3.3)	6 (27.3)	N/A^h^
Required RRT during follow-up (n (%))	118 (100)	8 (6.8)	1 (3.3)	1 (3.5)	1 (3.3)	5 (17.2)	N/A
**Death from any cause**	118 (100)						
Died during follow-up (n (%))		28 (23.7)	1 (3.3)	4 (13.8)	8 (26.7)	15 (51.7)	** < 0.001**
**Composite endpoints**	118 (100)						
Composite endpoint 1 during follow-up (n (%))^i^		49 (41.5)	8 (26.7)	9 (31.0)	12 (40.0)	20 (69.0)	**0.005**
Composite endpoint 2 during follow-up (n (%))^j^		39 (33.1)	5 (16.7)	6 (20.7)	9 (30.0)	19 (65.5)	** < 0.001**

^a^CKD-EPI, Chronic Kidney Disease-Epidemiology Collaboration; eGFR, estimated glomerular filtration rate; IQR, interquartile range; N/A, not applicable; Q1, quartile 1; Q2, quartile 2; Q3, quartile 3; Q4, quartile 4; RRT, renal replacement therapy; SD, standard deviation; sTNFR1, soluble tumour necrosis factor receptor-1.

^b^Values are given as n (%) for categorical variables, or mean ± SD for normally distributed continuous variables, unless otherwise indicated. Median [IQR] values are presented for continuous variables that are not normally distributed.

^c^Kruskall–Wallis test was used to assess for variation across the sTNFR1 quartiles in continuous variables that were not normally distributed.

^d^One-way between-groups ANOVA was used to assess for variation in normally distributed continuous variables across the sTNFR1 quartiles.

^e^n = 9 people were excluded (n = 7 with limited renal functional follow-up (< 1 year) due to initiation of renal replacement therapy or mortality and n = 2 with non-linear slopes of eGFR).

^f^n = 7 people with limited renal functional follow-up (< 1 year) due to initiation of renal replacement therapy or mortality were excluded.

^g^χ^2^ analysis was used to analyse for differences in categorical variables across the sTNFR1 quartiles.

^h^N/A indicates that the minimum expected cell frequency count for χ^2^ was not satisfied.

^i^Composite endpoint 1: ≥ 40% decrease in CKD-EPI eGFR, doubling of serum creatinine, renal replacement therapy, or mortality.

^j^Composite endpoint 2: doubling of serum creatinine, renal replacement therapy, or mortality.

### Univariate relationships of baseline uACR and sTNFR1 with renal and mortality endpoints

**Figure 1:**
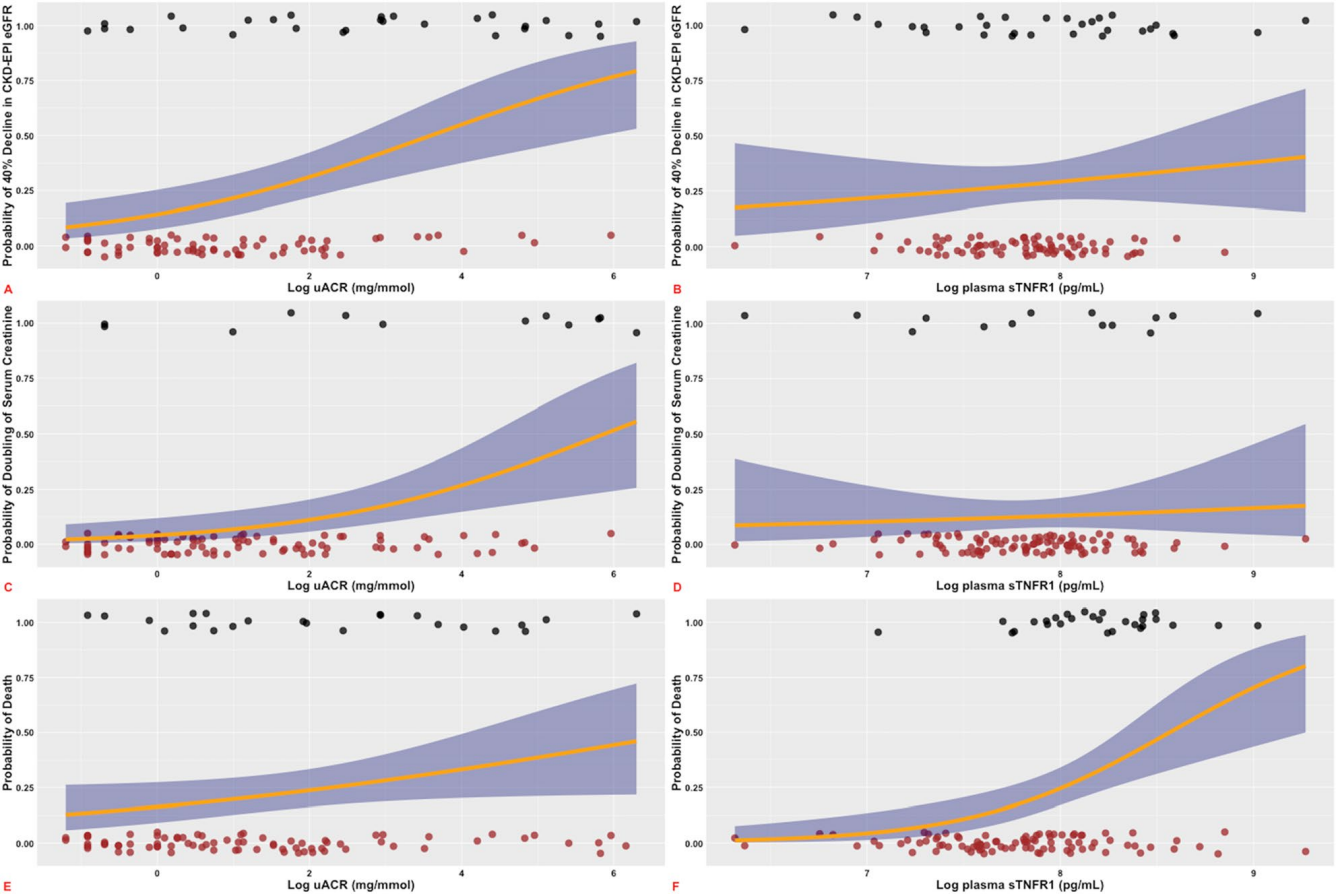
Univariate associations between uACR and plasma sTNFR1 with ≥ 40% decline in CKD-EPI eGFR, doubling of serum creatinine, and death. **A**: ≥ 40% decline in CKD-EPI eGFR with uACR; **B**: ≥ 40% decline in CKD-EPI eGFR with plasma sTNFR1; **C**: doubling of serum creatinine with uACR; **D**: doubling of serum creatinine with plasma sTNFR1; **E**: mortality with uACR; **F**: mortality with plasma sTNFR1. Individuals who did and did not develop the outcomes of interest are identified as 1 and 0 on the y-axis, respectively. Log-transformation of uACR and plasma sTNFR1 was performed prior to modelling. 95% confidence interval is represented by blue shading surrounding orange regression line of best fit.

**Figure 2:**
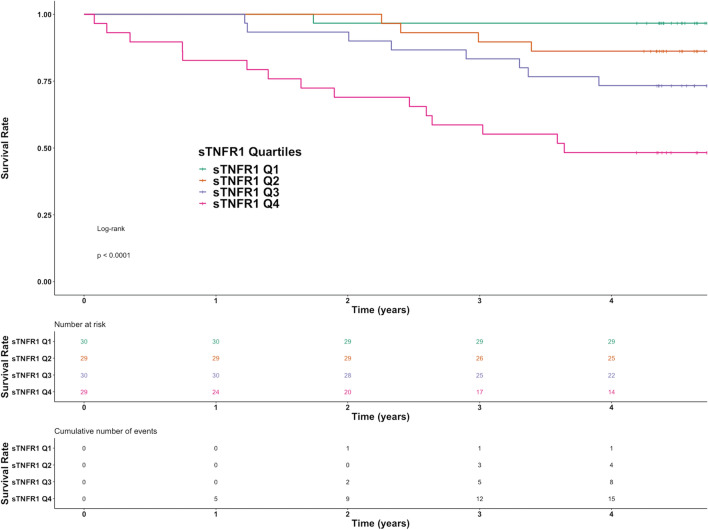
Unadjusted Kaplan–Meier survival plot stratified by plasma sTNFR1 quartile over the study period. Number of patients at risk of death and cumulative number of deaths are tabulated by plasma sTNFR1 quartile below the Kaplan–Meier plot. Log-rank p < 0.0001 for survival differences across the plasma sTNFR1 quartiles.

uACR strongly associated with ≥ 40% decline in CKD-EPI eGFR and doubling of serum creatinine by univariate binary logistic regression. Similarly, baseline uACR associated with the development of ESKD requiring renal replacement therapy during follow-up (p = 0.005), but more modestly predicted incident mortality (p = 0.05). Conversely, baseline sTNFR1 associated with death (p < 0.001) and the need for renal replacement therapy (p = 0.02), but not interim renal endpoints including ≥ 40% decline in CKD-EPI eGFR (p = 0.38) and doubling of serum creatinine (p = 0.65). Figure 1 presents univariate relationships of uACR and sTNFR1 with ≥ 40% decline in CKD-EPI eGFR, doubling of serum creatinine, and death, highlighting that uACR strongly predicted interim renal endpoints while sTNFR1 strongly predicted incident mortality. Conversely, baseline uACR weakly predicted incident mortality but sTNFR1 did not predict interim renal endpoints. Figure 2 presents an unadjusted Kaplan–Meier survival plot for the study cohort stratified by sTNFR1 quartile, demonstrating that there is an early and sustained increased incidence of death in sTNFR1 quartile 4 (log-rank p < 0.0001).

### uACR, but not sTNFR1, predicts accelerated renal functional decline (linear mixed-effects models)

Table 3 presents results of linear mixed-effects models of annual absolute and percentage changes in renal function according to baseline HbA_1c_, uACR and sTNFR1. Only uACR independently predicted subsequent accelerated renal functional decline. After adjusting for sTNFR1, per unit increase in the logarithm of baseline uACR, annual CKD-EPI eGFR decreased by − 0.56 mL/min/BSA/year, which corresponded to an annual loss of kidney function of 3.0% per year. Neither HbA_1c_ nor plasma sTNFR1 independently predicted a more negative eGFR slope trajectory in our study cohort. In a sensitivity analysis of annual changes in CKD-EPI eGFR restricted to those with ≥ 2 years of renal functional follow-up data, baseline uACR remained similarly predictive of subsequent renal functional decline (Supplementary Table 1). Baseline HbA_1c_ and sTNFR1 continued to show no association with subsequent loss of renal function.

**Table 3:** Annual changes in renal function according to baseline HbA_1c_, uACR and plasma sTNFR1 after adjustment for conventional risk factors for renal functional decline in the study cohort (n = 95)^a^.

Variable	Clinical model^b^			Clinical + sTNFR1 model^c^				Likelihood ratio p-value^d^
	Estimate	95% CI	p	Estimate		95% CI	p	
**Absolute change in renal function (mL/min/BSA/year)**								
**CKD-EPI eGFR**								0.51
HbA_1c_	− 0.04	− 0.10 to 0.02	0.24	− 0.03		− 0.10 to 0.03	0.28	
uACR	− 0.57	− 0.95 to − 0.19	**0.004**	− 0.56		− 0.94 to − 0.18	**0.004**	
sTNFR1	N/A	N/A	N/A	− 0.74		− 2.22 to 0.73	0.33	
**Percentage change in renal function (%/year)**								
**CKD-EPI eGFR**								0.24
HbA_1c_	− 0.15	− 0.37 to 0.08	0.20	− 0.13		− 0.35 to 0.09	0.25	
uACR	− 3.01	− 4.40 to − 1.64	** < 0.001**	− 2.97		− 4.34 to − 1.62	** < 0.001**	
sTNFR1	N/A	N/A	N/A	− 4.22		− 9.56 to 1.06	0.12	

^a^95% CI, 95% confidence interval; BSA, body surface area; CKD-EPI, Chronic Kidney Disease-Epidemiology Collaboration; eGFR, estimated glomerular filtration rate; HbA_1c_, glycated haemoglobin; sTNFR1, soluble tumour necrosis factor receptor-1; uACR, urine albumin-to-creatinine ratio.

^b^Clinical model: age, gender, diabetes duration, systolic blood pressure, HbA_1c_, CKD-EPI eGFR, uACR.

^c^Clinical + sTNFR1 model: clinical model + plasma sTNFR1.

^d^Clinical model versus clinical + sTNFR1 model.

### sTNFR1 predicts mortality but not interim renal endpoints (multivariable Cox proportional hazards regression and Fine-Gray models)

Table 4 presents results of multivariable Cox proportional hazards regression analyses for mortality, renal, and composite endpoints according to baseline HbA_1c_, uACR and sTNFR1.

**Table 4:** Cox proportional hazards regression of the risk of renal endpoints and mortality according to baseline HbA_1c_, uACR and plasma sTNFR1 after adjustment for conventional risk factors for renal functional decline in the study cohort^a^.

Variables	Clinical model^b^			Clinical + sTNFR1 model^c^			Likelihood ratio p-value^d^
	HR	95% CI	p	HR	95% CI	p	
** ≥ 40% decrease in CKD-EPI eGFR (n = 97)**							0.30
HbA_1c_	1.03	1.00–1.07	**0.049**	1.03	1.00–1.07	0.053	
uACR	1.49	1.17–1.90	**0.001**	1.49	1.16–1.90	**0.001**	
sTNFR1	N/A	N/A	N/A	0.63	0.27–1.47	0.29	
**Doubling of serum creatinine (n = 97)**							0.83
HbA_1c_	1.01	0.96–1.07	0.60	1.01	0.96–1.07	0.63	
uACR	2.01	1.34–3.01	** < 0.001**	2.00	1.34–3.00	** < 0.001**	
sTNFR1	N/A	N/A	N/A	0.88	0.26–2.90	0.83	
**Mortality (n = 101)**							**0.01**
HbA_1c_	1.03	1.00–1.06	0.10	1.02	0.99–1.05	0.24	
uACR	1.07	0.83–1.40	0.59	0.95	0.73–1.25	0.73	
sTNFR1	N/A	N/A	N/A	4.87	1.34–17.62	**0.02**	
**Composite endpoint 1**^e^** (n = 101)**							0.58
HbA_1c_	1.03	1.00–1.06	**0.03**	1.03	1.00–1.06	**0.03**	
uACR	1.35	1.11–1.63	**0.002**	1.34	1.11–1.63	**0.003**	
sTNFR1	N/A	N/A	N/A	1.24	0.58–2.66	0.58	
**Composite endpoint 2**^f^ **(n = 101)**							0.19
HbA_1c_	1.02	1.00–1.05	0.10	1.02	0.99–1.05	0.14	
uACR	1.29	1.05–1.59	**0.02**	1.27	1.03–1.57	**0.03**	
sTNFR1	N/A	N/A	N/A	1.80	0.72–4.48	0.21	

^a^95% CI, 95% confidence interval; CKD-EPI, Chronic Kidney Disease-Epidemiology Collaboration; eGFR, estimated glomerular filtration rate; HbA_1c_, glycated haemoglobin; HR, hazard ratio; N/A, not applicable; sTNFR1, soluble tumour necrosis factor receptor-1; uACR, urine albumin-to-creatinine ratio.

^b^Clinical model: age, gender, diabetes duration, systolic blood pressure, HbA_1c_, CKD-EPI eGFR, uACR.

^c^Clinical + sTNFR1 model: clinical model + plasma sTNFR1.

^d^Clinical model versus clinical + sTNFR1 model.

^e^Composite endpoint 1: ≥ 40% decrease in CKD-EPI eGFR, doubling of serum creatinine, renal replacement therapy, or mortality.

^f^Composite endpoint 2: doubling of serum creatinine, renal replacement therapy, or mortality.

**Figure 3:**
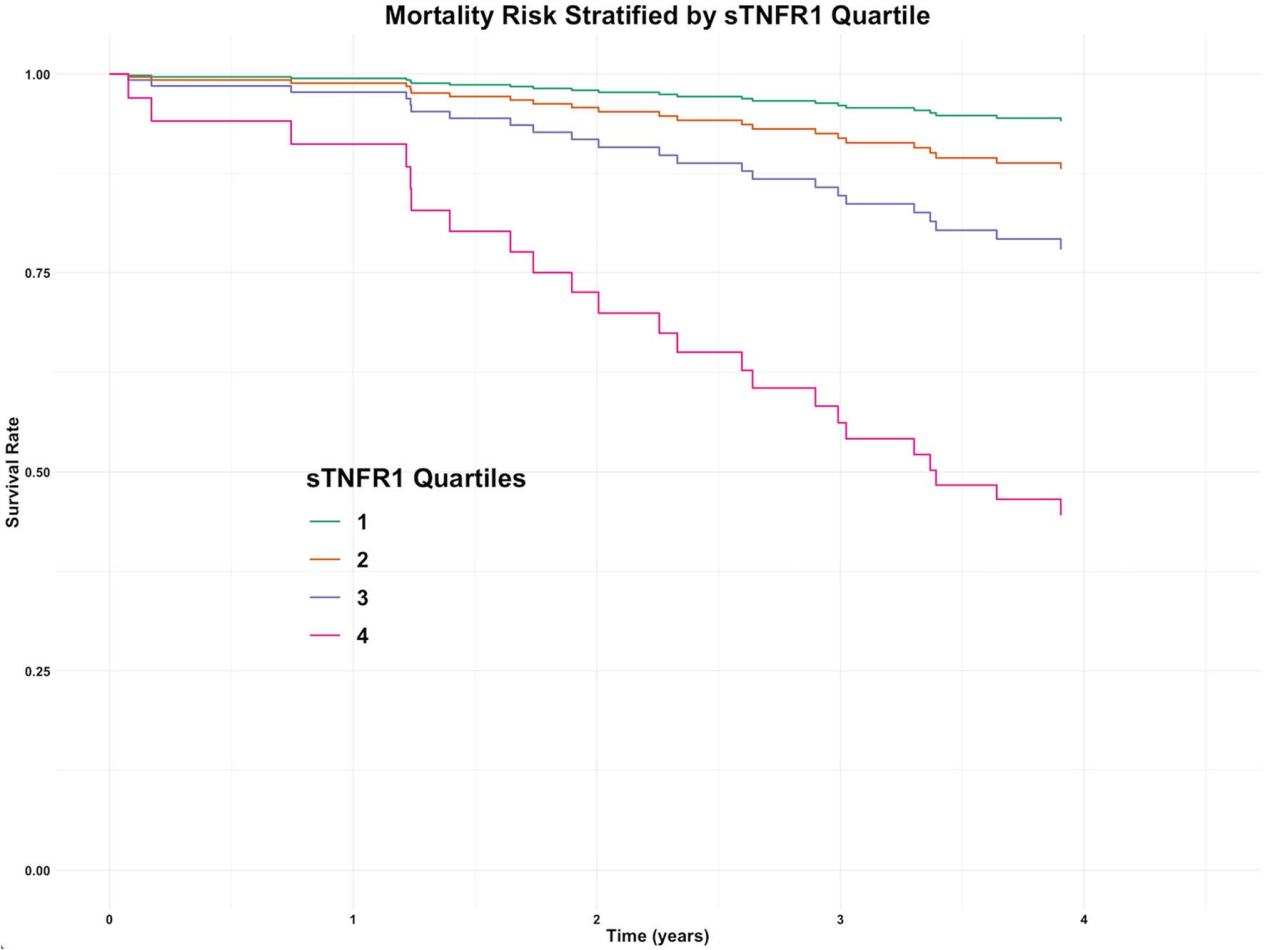
Survival plot of adjusted Cox proportional hazards model stratified by plasma sTNFR1 quartile during the study period. Cox model was adjusted for: age, gender, diabetes duration, systolic blood pressure, HbA_1c_, CKD-EPI eGFR, and uACR.

Elevated sTNFR1, but not uACR, increased the risk of incident mortality (HR 4.87, p = 0.02). Elevated uACR, but not sTNFR1, increased the risk of ≥ 40% decline in CKD-EPI eGFR (HR 1.49, p = 0.001) and doubling of serum creatinine (HR 2.00, p < 0.001). A Cox proportional hazards regression model adjusted for clinical variables and sTNFR1 quartile is presented in Fig. 3, reinforcing the high incident mortality rates over 4 years in people in quartile 4 of sTNFR1.

Consequently, increased baseline uACR (HR 1.34, p = 0.003), but not sTNFR1, increased the risk of a composite endpoint of ≥ 40% decline in CKD-EPI eGFR, doubling of serum creatinine, renal replacement therapy, or death. Baseline HbA_1c_ did not predict individual renal and mortality outcomes during follow-up. However, increased baseline HbA_1c_ did independently predict the development of a composite endpoint of ≥ 40% decline in CKD-EPI eGFR, doubling of serum creatinine, renal replacement therapy, or death (p = 0.03), albeit in a much weaker fashion (HR 1.03) than baseline uACR. The strong univariate association between sTNFR1 and mortality persisted on multivariable regression, while associations with interim renal endpoints were not evident when sTNFR1 was considered in addition to other clinical variables. sTNFR1 did not improve model performance for prediction of ≥ 40% decline in CKD-EPI eGFR, doubling of serum creatinine, or composite renal and mortality endpoints. However, sTNFR1 did improve prediction of incident mortality by Cox regression models (p = 0.01). In a sensitivity analysis, results from multivariable Cox proportional hazards regression were validated using multivariable logistic regression adjusting for the same clinical covariates, with essentially identical findings (Supplementary Table 2). An additional sensitivity analysis which restricted multivariate Cox and logistic regression analyses to those with ≥ 2 years of renal functional follow-up data similarly identified baseline uACR, but not plasma sTNFR1, as strongly predictive of interim renal endpoints (Supplementary Table 3).

**Figure 4:**
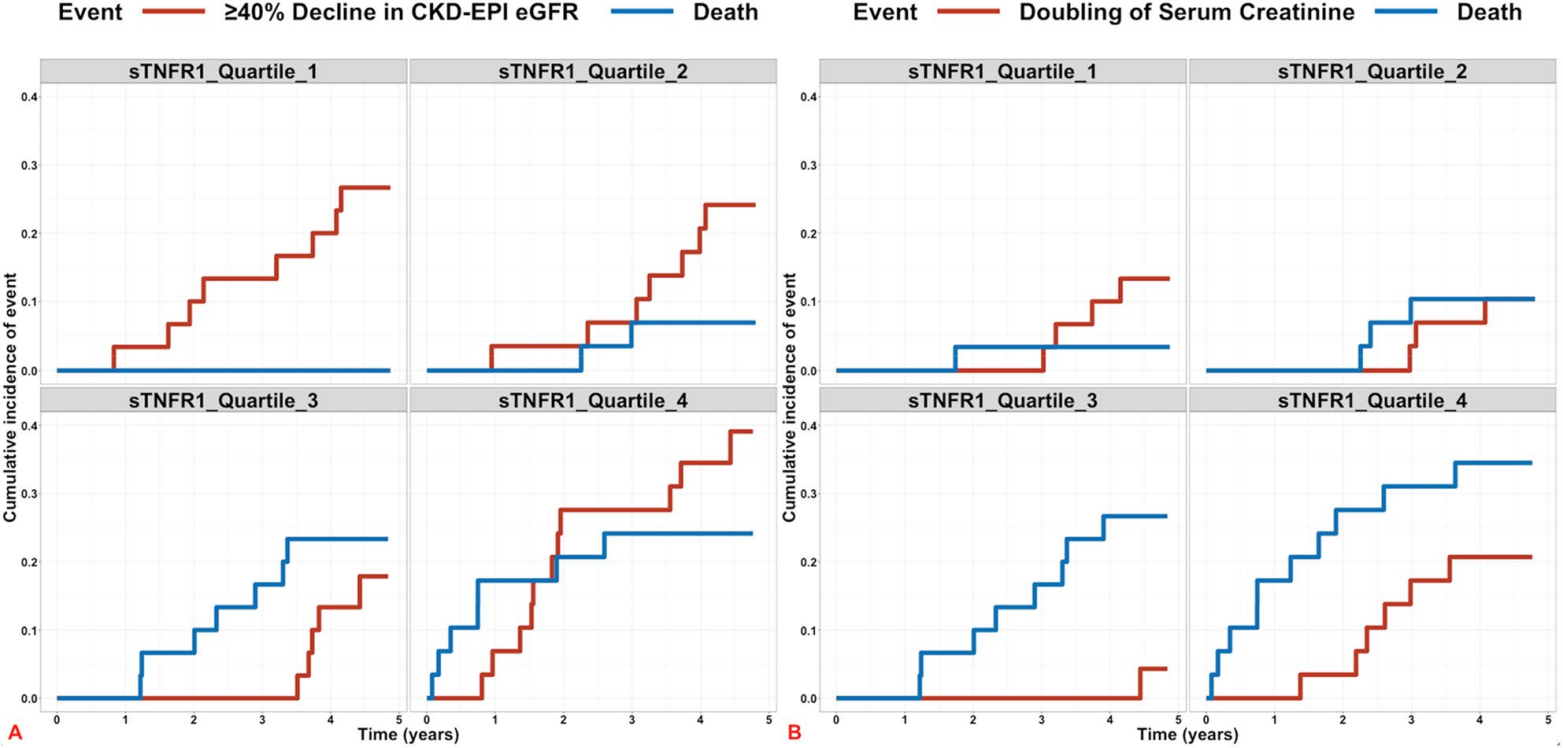
Cumulative incidence of  ≥ 40% decline in CKD-EPI eGFR and death (**A**) and doubling of serum creatinine and death (**B**) stratified by plasma sTNFR1 quartile over the study period. Cumulative incidence of events of interest (≥ 40% decline in CKD-EPI eGFR and doubling of serum creatinine) and the competing risk of death were calculated using the cuminc function in the R package cmprsk. Cumulative incidence plots were generated using ggcompetingrisks from the R package survminer. Panels are labelled by baseline plasma sTNFR1 quartiles 1, 2, 3, and 4, respectively. The cumulative incidence of renal outcomes over the study period is depicted by the red lines; the cumulative incidence of the competing risk of death is represented by the blue lines. In plasma sTNFR1 quartiles 3 and 4, the competing risk of death occurred more commonly than renal outcomes including ≥ 40% decline in CKD-EPI eGFR and doubling of serum creatinine.

Considering all-cause mortality as a competing risk, subdistribution HRs for the risk of ≥ 40% decline in CKD-EPI eGFR and doubling of serum creatinine are presented in Table 5. Similar to Cox models, an increased risk of renal endpoints was observed with elevated uACR: subdistribution HRs 1.46 (p = 0.001) and 1.99 (p < 0.001) for ≥ 40% decline in CKD-EPI eGFR and doubling of serum creatinine, respectively. sTNFR1 did not independently predict interim renal endpoints. Figure 4 presents the cumulative incidence of renal endpoints and the competing risk of all-cause mortality. The incidence of death increased linearly and significantly across baseline sTNFR1 quartiles over the study period. Increases in the incidence of interim renal endpoints across baseline sTNFR1 quartiles were much smaller in magnitude and did not reach statistical significance. P-values for differences in the incidence of ≥ 40% decline in CKD-EPI eGFR and death across sTNFR1 quartiles were 0.22 and 0.01, respectively. P-values for differences in the incidence of doubling of serum creatinine and death across sTNFR1 quartiles were 0.19 and 0.006, respectively.

**Table 5:** Fine-Gray model regression of the risk of renal endpoints according to baseline HbA_1c_, uACR and plasma sTNFR1 after adjustment for conventional risk factors for renal functional decline and the competing risk of death in the study cohort^a,b^.

Variables	Clinical model^c^			Clinical + sTNFR1 model^d^		
	HR	95% CI	p	HR	95% CI	p
** ≥ 40% decrease in CKD-EPI eGFR (n = 101)**						
HbA_1c_	1.03	1.00–1.06	**0.05**	1.03	0.99–1.06	0.11
uACR	1.44	1.15–1.81	**0.002**	1.46	1.17–1.83	**0.001**
sTNFR1	N/A	N/A	N/A	0.49	0.22–1.13	0.10
**Doubling of serum creatinine (n = 101)**						
HbA_1c_	1.02	0.98–1.05	0.32	1.01	0.98–1.05	0.47
uACR	1.98	1.34–2.93	**0.001**	1.99	1.35–2.92	** < 0.001**
sTNFR1	N/A	N/A	N/A	0.68	0.30–1.58	0.37

^a^95% CI, 95% confidence interval; CKD-EPI, Chronic Kidney Disease-Epidemiology Collaboration; eGFR, estimated glomerular filtration rate; HbA_1c_, glycated haemoglobin; HR, hazard ratio; N/A, not applicable; sTNFR1, soluble tumour necrosis factor receptor-1; uACR, urine albumin-to-creatinine ratio.

^b^Competing risk = all-cause mortality.

^c^Clinical model: age, gender, diabetes duration, systolic blood pressure, HbA_1c_, CKD-EPI eGFR, uACR.

^d^Clinical + sTNFR1 model: clinical model + plasma sTNFR1.

## Discussion

This study provides insight into the complementary predictive performance of plasma sTNFR1 and uACR in the identification of subjects at high-risk of mortality and accelerated renal functional decline in older persons with longstanding type 2 diabetes and eGFR ≤ 60 mL/min/BSA in real-world clinical practice. We demonstrate that mortality rates are high in this population, and are strongly and independently predicted by sTNFR1. sTNFR1 provided no incremental benefit to conventional clinical variables in the identification of subjects at increased risk of accelerated renal functional decline.

Our study reaffirms the strong prognostication of accelerated renal functional decline by baseline levels of albuminuria in people with diabetes and CKD^26^. That sTNFR1 provided no incremental benefit in the identification of such patients in our dataset was somewhat surprising and contrary to the findings of Saulnier et al. who demonstrated that in a French cohort of patients with type 2 diabetes (SURDIAGENE), adjusting for the same clinical parameters used herein, plasma sTNFR1 independently identified an increased risk of ≥ 40% decline in eGFR and decline of GFR < − 5 mL/min/BSA/year^13^. However, mean age (64 versus 74 years) and diabetes duration (14 versus 16 years) were lower while mean eGFR (76 versus 42 mL/min/BSA) was higher in the SURDIAGENE study than in our cohort^13^.

In later CKD stages, is it possible that a combination of improved clinical prognostication of renal functional decline with routine variables (supported by the accuracy of the Kidney Failure Risk Equation in this setting) coupled with higher cardiovascular mortality diminishes the ability of sTNFR1 to independently identify people at risk of renal functional loss^27,28^. Median annual loss of CKD-EPI eGFR was relatively low in our cohort at − 2 mL/min/BSA/year, compared with − 5.6 mL/min/BSA/year in an Irish cohort study of patients with type 2 diabetic kidney disease (mean baseline eGFR 47 mL/min/BSA)^5^. Additionally, the study cohort had a relatively low burden of proteinuria, with median uACR for the study cohort being at the threshold for diagnosis of microalbuminuria (3.0 mg/mmol). Our study cohort was also elderly (mean age 74 years) with a high burden of coronary and peripheral artery disease (18% each). Non-proteinuric or minimally proteinuric diabetic kidney disease appears to be more common in those with prior cardiovascular events, and such patients are at higher risk of future cardiovascular than renal events^29^. Therefore, it is plausible that our elderly cohort with a high burden of established cardiovascular disease, minimal proteinuria, and slow rate of renal functional decline were primed for cardiovascular rather than renal events, which diminished statistical power to detect relationships between plasma sTNFR1 and progressive renal disease. Survival bias may also explain the lack of association between plasma sTNFR1 and progressive renal disease in our study, given that the population is older and had survived with CKD for longer than other younger cohorts in which sTNFR1 has been evaluated to date^30^. In a subset of the SURDIAGENE cohort (mean age 70 years, diabetes duration 18 years, and eGFR 49 mL/min/BSA) more similar to our study population, sTNFR1 independently predicted a composite of doubling of serum creatinine or ESKD and also all-cause mortality^12^. Individual renal endpoints were not reported therein.

Management of diabetic kidney disease centres on intensification of blood pressure control and renin-angiotensin-aldosterone system blockade to control proteinuria^31^. Currently, SGLT2is are relatively contraindicated in people with diabetes and eGFR ≤ 45 mL/min/BSA due to lack of glycaemic efficacy^32^. New therapies which improve outcomes for people with type 2 diabetes and more advanced CKD are required. Intentional weight loss strategies, including metabolic surgery, and mesenchymal stem cells hold promise in this regard^6,7,33^. Integrated diabetes and nephrology care at later CKD stages may also improve patient outcomes, particularly for people with diabetes and additional CKD aetiologies^5^, and should be considered to lower cardiovascular and renal risk in those with higher sTNFR1 values.

The predominance of white Caucasians in our cohort limits the applicability of our findings to adults from other racial and ethnic backgrounds, but there is no evidence at present to suggest that sTNFR1 behaves differently in patients from other racial backgrounds. Our study cohort was relatively small which may have limited statistical power to detect significant relationships between baseline plasma sTNFR1 and renal outcomes. However, our patient population was well characterised clinically and analyses of trends in renal function were made on the basis of a median of 14 eGFR values over mean 3.5 years of follow-up. Additionally, event rates of renal and mortality outcomes were high in our study cohort which improved statistical power, while study findings were replicated using several multivariate regression modelling approaches.

Baseline HbA_1c_ values may have been falsely lowered by anaemia accompanying CKD, although mean haemoglobin for the study cohort was 12.7 g/dL and no patients were treated with an erythropoietin-stimulating agent. Future studies assessing the prognostic value of biomarkers in people with diabetes and CKD should consider adjusting multivariate analyses with alternative indicators of glycaemic control, such as glycated albumin^34^. Our study does lacks detail on the prevalence of additional CKD aetiologies other than diabetes. However, given the median diabetes duration of almost 16 years in the study cohort, it is reasonable to assume that most patients had CKD as a sequel to their diabetes. The generalisability of our findings to patients without diabetes is unknown and should be investigated in future studies.

## Conclusions

In conclusion, mortality rates were high (over 50% over 4-year follow-up) in people with type 2 diabetes, CKD, and elevated plasma sTNFR1. uACR, but not sTNFR1, identified patients with subsequent accelerated renal functional decline. Conversely, increased sTNFR1 identified subjects at significantly increased risk of all-cause mortality but not accelerated renal functional decline. Thus, in this elderly cohort with longstanding type 2 diabetes and established CKD, plasma sTNFR1 and uACR provided complementary risk prediction of mortality and progressive renal disease, respectively.

## Supplementary information

Supplementary information.

Supplementary Table 1.

Supplementary Table 2.

Supplementary Table 3.

## Data Availability

The datasets used during the current study are available from the corresponding author on reasonable request.

## Abbreviations

ACE: Angiotensin-converting enzyme
ARB: Angiotensin-II receptor blocker
BSA: Body surface area
CKD: Chronic kidney disease
CKD-EPI: Chronic kidney disease-epidemiology collaboration
DKD: Diabetic kidney disease
eGFR: Estimated glomerular filtration rate
ESA: Erythropoietin-stimulating agent
ESKD: End-stage kidney disease
GLP1RA: Glucagon-like peptide-1 receptor analogue
HbA_1c_: Glycated haemoglobin
HR: Hazard ratio
IQR: Interquartile range
OR: Odds ratio
Q1: Quartile 1
Q2: Quartile 2
Q3: Quartile 3
Q4: Quartile 4
RRT: Renal replacement therapy
SD: Standard deviation
SGLT2i: Sodium-glucose co-transporter-2 inhibitor
sTNFR1: Soluble tumour necrosis factor receptor-1
uACR: Urine albumin-to-creatinine ratio
