# Supplementary material for: Parallel assessment of albuminuria and plasma sTNFR1 in people with type 2 diabetes and advanced chronic kidney disease provides accurate prognostication of the risks of renal decline and death
Source: Sci Rep. 2020 Sep 9;10:14852. doi: 10.1038/s41598-020-71684-6 (PMC7481247; doi:10.1038/s41598-020-71684-6)
Supplement: Supplementary file 1 — Supplementary information. [file 41598_2020_71684_MOESM1_ESM.docx]

**Parallel Assessment of Albuminuria and Plasma sTNFR1 in People with Type 2 Diabetes and Advanced Chronic Kidney Disease Provides Accurate Prognostication of the Risks of Renal Decline and Death.**

William P. Martin^1^, Colm Tuohy^1^, Alison Doody^1^, Sabrina Jackson^1^, Ronan J. Canavan^2^, David Slattery^2^, Patrick J. Twomey^3^, Malachi J. McKenna^2^, Carel W. le Roux^1,4,5^ and Neil G. Docherty^1,4^.

^1^Diabetes Complications Research Centre, School of Medicine, Conway Institute, University College Dublin, Ireland.

^2^Department of Endocrinology, St. Vincent’s University Hospital, Elm Park, Dublin 4, Ireland.

^3^Department of Clinical Chemistry, St. Vincent’s University Hospital, Elm Park, Dublin 4, Ireland.

^4^Institute of Clinical Sciences, Sahlgrenska Academy, University of Gothenburg, Sweden.

^5^Division of Investigative Science, Imperial College London, United Kingdom.

**Correspondence to:**

Dr. Neil Docherty

Diabetes Complications Research Centre

School of Medicine

Conway Institute of Biomolecular and Biomedical Science

University College Dublin

Belfield, Dublin 4

Ireland

+353-1716-6877

[neil.docherty@ucd.ie](mailto:neil.docherty@ucd.ie)

@UCDDCRC

**Supplementary Table S1. Annual Changes in Renal Function in Those with ≥2 Years’ Renal Functional Follow-Up Data According to Baseline HbA_1c_, uACR and Plasma sTNFR1 After Adjustment for Conventional Risk Factors for Renal Functional Decline in the Study Cohort (n=87).^a^**

| **Variable** | **Clinical model^b^** | | | | **Clinical + sTNFR1 model^c^** | | | **Likelihood ratio p-value^d^** |
| --- | --- | --- | --- | --- | --- | --- | --- | --- |
|  | **Estimate** | **95% CI** | **p** | **Estimate** | | **95% CI** | **p** |  |
| **Absolute change in renal function (mL/min/BSA/year)** |  | | | | | | | |
| **CKD-EPI eGFR** |  |  |  |  | |  |  | 0.53 |
| HbA_1c_ | -0.03 | -0.10 – 0.03 | 0.27 | -0.03 | | -0.09 – 0.03 | 0.30 |  |
| uACR | -0.52 | -0.91 - -0.13 | **0.01** | -0.52 | | -0.91­ - -0.13 | **0.009** |  |
| sTNFR1 | N/A | N/A | N/A | -0.75 | | -2.26 – 0.74 | 0.32 |  |
| **Percentage change in renal function (%/year)** |  | | | | | | | |
| **CKD-EPI eGFR** |  |  |  |  | |  |  | 0.37 |
| HbA_1c_ | -0.13 | -0.35 – 0.08 | 0.22 | -0.12 | | -0.34 – 0.09 | 0.26 |  |
| uACR | -2.66 | -4.04 - -1.30 | **<0.001** | -2.66 | | -4.02 - -1.31 | **<0.001** |  |
| sTNFR1 | N/A | N/A | N/A | -3.50 | | -8.72 – 1.69 | 0.19 |  |

^a^95% CI = 95% confidence interval; BSA = body surface area; CKD-EPI = Chronic Kidney Disease-Epidemiology Collaboration; eGFR = estimated glomerular filtration rate; HbA_1c_ = glycated haemoglobin; sTNFR1 = soluble tumour necrosis factor receptor-1; uACR = urine albumin-to-creatinine ratio.

^b^Clinical model: age, gender, diabetes duration, systolic blood pressure, HbA_1c_, CKD-EPI eGFR, uACR.

^c^Clinical + sTNFR1 model: clinical model + plasma sTNFR1.

^d^Clinical model versus clinical + sTNFR1 model.

**Supplementary Table S2. Logistic Regression of the Risk of Renal Endpoints and Mortality According to Baseline HbA_1c_, uACR and Plasma sTNFR1 after Adjustment for Conventional Risk Factors for Renal Functional Decline in the Study Cohort.^a^**

| **Variables** | **Clinical model^b^** | | | **Clinical + sTNFR1 model^c^** | | | **Likelihood ratio p-value^d^** |
| --- | --- | --- | --- | --- | --- | --- | --- |
|  | **OR** | **95% CI** | **p** | **OR** | **95% CI** | **p** |  |
| **≥40% decrease in CKD-EPI eGFR (n=97)** |  | | | | | | 0.18 |
| HbA_1c_ | 1.02 | 0.98-1.07 | 0.35 | 1.03 | 0.98-1.08 | 0.27 |  |
| uACR | 1.62 | 1.19-2.28 | **0.003** | 1.66 | 1.21-2.38 | **0.003** |  |
| sTNFR1 | N/A | N/A | N/A | 0.42 | 0.11-1.47 | 0.19 |  |
| **Doubling of serum creatinine (n=97)** |  | | | | | | 0.47 |
| HbA_1c_ | 1.01 | 0.94-1.07 | 0.84 | 1.01 | 0.94-1.07 | 0.88 |  |
| uACR | 2.15 | 1.38-3.65 | **0.002** | 2.18 | 1.40-3.78 | **0.002** |  |
| sTNFR1 | N/A | N/A | N/A | 0.58 | 0.12-2.63 | 0.48 |  |
| **Mortality (n=101)** |  | | | | | | **0.01** |
| HbA_1c_ | 1.06 | 1.00-1.12 | 0.05 | 1.05 | 0.99-1.11 | 0.09 |  |
| uACR | 1.08 | 0.78-1.50 | 0.65 | 0.96 | 0.67-1.37 | 0.84 |  |
| sTNFR1 | N/A | N/A | N/A | 7.93 | 1.57-52.8 | **0.02** |  |
| **Composite endpoint 1^e^ (n=101)** |  | | | | | | 0.79 |
| HbA_1c_ | 1.04 | 0.99-1.09 | 0.14 | 1.04 | 0.99-1.09 | 0.15 |  |
| uACR | 1.55 | 1.17-2.13 | **0.003** | 1.55 | 1.17-2.13 | **0.004** |  |
| sTNFR1 | N/A | N/A | N/A | 1.16 | 0.38-3.49 | 0.79 |  |
| **Composite endpoint 2^f^ (n=101)** |  | | | | | | 0.21 |
| HbA_1c_ | 1.04 | 1.00-1.09 | 0.09 | 1.04 | 0.99-1.09 | 0.10 |  |
| uACR | 1.42 | 1.07-1.92 | **0.02** | 1.40 | 1.06-1.90 | **0.02** |  |
| sTNFR1 | N/A | N/A | N/A | 2.04 | 0.67-6.62 | 0.22 |  |

^a^95% CI = 95% confidence interval; CKD-EPI = Chronic Kidney Disease-Epidemiology Collaboration; eGFR = estimated glomerular filtration rate; HbA_1c_ = glycated haemoglobin; N/A = not applicable; OR = odds ratio; sTNFR1 = soluble tumour necrosis factor receptor-1; uACR = urine albumin-to-creatinine ratio.

^b^Clinical model: age, gender, diabetes duration, systolic blood pressure, HbA_1c_, CKD-EPI eGFR, uACR.

^c^Clinical + sTNFR1 model: clinical model + plasma sTNFR1.

^d^Clinical model versus clinical + sTNFR1 model.

^e^Composite endpoint 1: ≥40% decrease in CKD-EPI eGFR, doubling of serum creatinine, renal replacement therapy, or mortality.

^f^Composite endpoint 2: doubling of serum creatinine, renal replacement therapy, or mortality.

**Supplementary Table S3. Cox Proportional Hazards and Logistic Regression of the Risk of Renal Endpoints and Mortality in Those with ≥2 Years’ Renal Functional Follow-Up Data According to Baseline HbA_1c_, uACR and Plasma sTNFR1 after Adjustment for Conventional Risk Factors for Renal Functional Decline in the Study Cohort (n=87).^a^**

| **Variables** | **Cox proportional hazards regression** | | | | | | |
| --- | --- | --- | --- | --- | --- | --- | --- |
|  | **Clinical model^b^** | | | **Clinical + sTNFR1 model^c^** | | | **Likelihood ratio p-value^d^** |
|  | **HR** | **95% CI** | **p** | **HR** | **95% CI** | **p** |  |
| **≥40% decrease in CKD-EPI eGFR** |  | | | | | | 0.33 |
| HbA_1c_ | 1.04 | 1.00-1.08 | 0.07 | 1.04 | 1.00-1.08 | 0.07 |  |
| uACR | 1.56 | 1.19-2.05 | **0.001** | 1.55 | 1.18-2.03 | **0.002** |  |
| sTNFR1 | N/A | N/A | N/A | 0.64 | 0.26-1.56 | 0.33 |  |
| **Doubling of serum creatinine** |  | | | | | | 0.52 |
| HbA_1c_ | 1.02 | 0.96-1.08 | 0.57 | 1.01 | 0.96-1.08 | 0.64 |  |
| uACR | 1.90 | 1.26-2.87 | **0.002** | 1.87 | 1.24-2.84 | **0.003** |  |
| sTNFR1 | N/A | N/A | N/A | 0.67 | 0.20-2.25 | 0.51 |  |
|  | **Logistic regression** | | | | | | |
|  | **Clinical model^b^** | | | **Clinical + sTNFR1 model^c^** | | |  |
|  | **OR** | **95% CI** | **p** | **OR** | **95% CI** | **p** |  |
| **≥40% decrease in CKD-EPI eGFR** |  |  |  |  |  |  | 0.29 |
| HbA_1c_ | 1.02 | 0.97-1.07 | 0.39 | 1.02 | 0.98-1.08 | 0.34 |  |
| uACR | 1.64 | 1.18-2.39 | **0.005** | 1.66 | 1.18-2.44 | **0.006** |  |
| sTNFR1 | N/A | N/A | N/A | 0.49 | 0.12-1.81 | 0.29 |  |
| **Doubling of serum creatinine** |  |  |  |  |  |  | 0.35 |
| HbA_1c_ | 1.01 | 0.94-1.07 | 0.86 | 1.00 | 0.94-1.07 | 0.89 |  |
| uACR | 2.09 | 1.31-3.69 | **0.004** | 2.08 | 1.30-3.69 | **0.005** |  |
| sTNFR1 | N/A | N/A | N/A | 0.46 | 0.08-2.30 | 0.35 |  |

^a^95% CI = 95% confidence interval; CKD-EPI = Chronic Kidney Disease-Epidemiology Collaboration; eGFR = estimated glomerular filtration rate; HbA_1c_ = glycated haemoglobin; HR = hazard ratio; N/A = not applicable; OR = odds ratio; sTNFR1 = soluble tumour necrosis factor receptor-1; uACR = urine albumin-to-creatinine ratio.

^b^Clinical model: age, gender, diabetes duration, systolic blood pressure, HbA_1c_, CKD-EPI eGFR, uACR.

^c^Clinical + sTNFR1 model: clinical model + plasma sTNFR1.

^d^Clinical model versus clinical + sTNFR1 model.

^e^Composite endpoint 1: ≥40% decrease in CKD-EPI eGFR, doubling of serum creatinine, renal replacement therapy, or mortality.

^f^Composite endpoint 2: doubling of serum creatinine, renal replacement therapy, or mortality.
