# Supplementary material for: Parallel assessment of albuminuria and plasma sTNFR1 in people with type 2 diabetes and advanced chronic kidney disease provides accurate prognostication of the risks of renal decline and death
Source: Sci Rep. 2020 Sep 9;10:14852. doi: 10.1038/s41598-020-71684-6 (PMC7481247; doi:10.1038/s41598-020-71684-6)
Supplement: Supplementary file 2 — Supplementary Table 1. [file 41598_2020_71684_MOESM2_ESM.docx]

**Supplementary Table S1. Annual Changes in Renal Function in Those with ≥2 Years’ Renal Functional Follow-Up Data According to Baseline HbA_1c_, uACR and Plasma sTNFR1 After Adjustment for Conventional Risk Factors for Renal Functional Decline in the Study Cohort (n=87).^a^**

| **Variable** | **Clinical model^b^** | | | | **Clinical + sTNFR1 model^c^** | | | **Likelihood ratio p-value^d^** |
| --- | --- | --- | --- | --- | --- | --- | --- | --- |
|  | **Estimate** | **95% CI** | **p** | **Estimate** | | **95% CI** | **p** |  |
| **Absolute change in renal function (mL/min/BSA/year)** |  | | | | | | | |
| **CKD-EPI eGFR** |  |  |  |  | |  |  | 0.53 |
| HbA_1c_ | -0.03 | -0.10 – 0.03 | 0.27 | -0.03 | | -0.09 – 0.03 | 0.30 |  |
| uACR | -0.52 | -0.91 - -0.13 | **0.01** | -0.52 | | -0.91­ - -0.13 | **0.009** |  |
| sTNFR1 | N/A | N/A | N/A | -0.75 | | -2.26 – 0.74 | 0.32 |  |
| **Percentage change in renal function (%/year)** |  | | | | | | | |
| **CKD-EPI eGFR** |  |  |  |  | |  |  | 0.37 |
| HbA_1c_ | -0.13 | -0.35 – 0.08 | 0.22 | -0.12 | | -0.34 – 0.09 | 0.26 |  |
| uACR | -2.66 | -4.04 - -1.30 | **<0.001** | -2.66 | | -4.02 - -1.31 | **<0.001** |  |
| sTNFR1 | N/A | N/A | N/A | -3.50 | | -8.72 – 1.69 | 0.19 |  |

^a^95% CI = 95% confidence interval; BSA = body surface area; CKD-EPI = Chronic Kidney Disease-Epidemiology Collaboration; eGFR = estimated glomerular filtration rate; HbA_1c_ = glycated haemoglobin; sTNFR1 = soluble tumour necrosis factor receptor-1; uACR = urine albumin-to-creatinine ratio.

^b^Clinical model: age, gender, diabetes duration, systolic blood pressure, HbA_1c_, CKD-EPI eGFR, uACR.

^c^Clinical + sTNFR1 model: clinical model + plasma sTNFR1.

^d^Clinical model versus clinical + sTNFR1 model.
