# Supplementary material for: Parallel assessment of albuminuria and plasma sTNFR1 in people with type 2 diabetes and advanced chronic kidney disease provides accurate prognostication of the risks of renal decline and death
Source: Sci Rep. 2020 Sep 9;10:14852. doi: 10.1038/s41598-020-71684-6 (PMC7481247; doi:10.1038/s41598-020-71684-6)
Supplement: Supplementary file 3 — Supplementary Table 2. [file 41598_2020_71684_MOESM3_ESM.docx]

**Supplementary Table S2. Logistic Regression of the Risk of Renal Endpoints and Mortality According to Baseline HbA_1c_, uACR and Plasma sTNFR1 after Adjustment for Conventional Risk Factors for Renal Functional Decline in the Study Cohort.^a^**

| **Variables** | **Clinical model^b^** | | | **Clinical + sTNFR1 model^c^** | | | **Likelihood ratio p-value^d^** |
| --- | --- | --- | --- | --- | --- | --- | --- |
|  | **OR** | **95% CI** | **p** | **OR** | **95% CI** | **p** |  |
| **≥40% decrease in CKD-EPI eGFR (n=97)** |  | | | | | | 0.18 |
| HbA_1c_ | 1.02 | 0.98-1.07 | 0.35 | 1.03 | 0.98-1.08 | 0.27 |  |
| uACR | 1.62 | 1.19-2.28 | **0.003** | 1.66 | 1.21-2.38 | **0.003** |  |
| sTNFR1 | N/A | N/A | N/A | 0.42 | 0.11-1.47 | 0.19 |  |
| **Doubling of serum creatinine (n=97)** |  | | | | | | 0.47 |
| HbA_1c_ | 1.01 | 0.94-1.07 | 0.84 | 1.01 | 0.94-1.07 | 0.88 |  |
| uACR | 2.15 | 1.38-3.65 | **0.002** | 2.18 | 1.40-3.78 | **0.002** |  |
| sTNFR1 | N/A | N/A | N/A | 0.58 | 0.12-2.63 | 0.48 |  |
| **Mortality (n=101)** |  | | | | | | **0.01** |
| HbA_1c_ | 1.06 | 1.00-1.12 | 0.05 | 1.05 | 0.99-1.11 | 0.09 |  |
| uACR | 1.08 | 0.78-1.50 | 0.65 | 0.96 | 0.67-1.37 | 0.84 |  |
| sTNFR1 | N/A | N/A | N/A | 7.93 | 1.57-52.8 | **0.02** |  |
| **Composite endpoint 1^e^ (n=101)** |  | | | | | | 0.79 |
| HbA_1c_ | 1.04 | 0.99-1.09 | 0.14 | 1.04 | 0.99-1.09 | 0.15 |  |
| uACR | 1.55 | 1.17-2.13 | **0.003** | 1.55 | 1.17-2.13 | **0.004** |  |
| sTNFR1 | N/A | N/A | N/A | 1.16 | 0.38-3.49 | 0.79 |  |
| **Composite endpoint 2^f^ (n=101)** |  | | | | | | 0.21 |
| HbA_1c_ | 1.04 | 1.00-1.09 | 0.09 | 1.04 | 0.99-1.09 | 0.10 |  |
| uACR | 1.42 | 1.07-1.92 | **0.02** | 1.40 | 1.06-1.90 | **0.02** |  |
| sTNFR1 | N/A | N/A | N/A | 2.04 | 0.67-6.62 | 0.22 |  |

^a^95% CI = 95% confidence interval; CKD-EPI = Chronic Kidney Disease-Epidemiology Collaboration; eGFR = estimated glomerular filtration rate; HbA_1c_ = glycated haemoglobin; N/A = not applicable; OR = odds ratio; sTNFR1 = soluble tumour necrosis factor receptor-1; uACR = urine albumin-to-creatinine ratio.

^b^Clinical model: age, gender, diabetes duration, systolic blood pressure, HbA_1c_, CKD-EPI eGFR, uACR.

^c^Clinical + sTNFR1 model: clinical model + plasma sTNFR1.

^d^Clinical model versus clinical + sTNFR1 model.

^e^Composite endpoint 1: ≥40% decrease in CKD-EPI eGFR, doubling of serum creatinine, renal replacement therapy, or mortality.

^f^Composite endpoint 2: doubling of serum creatinine, renal replacement therapy, or mortality.
