# Supplementary material for: Parallel assessment of albuminuria and plasma sTNFR1 in people with type 2 diabetes and advanced chronic kidney disease provides accurate prognostication of the risks of renal decline and death
Source: Sci Rep. 2020 Sep 9;10:14852. doi: 10.1038/s41598-020-71684-6 (PMC7481247; doi:10.1038/s41598-020-71684-6)
Supplement: Supplementary file 4 — Supplementary Table 3. [file 41598_2020_71684_MOESM4_ESM.docx]

**Supplementary Table S3. Cox Proportional Hazards and Logistic Regression of the Risk of Renal Endpoints and Mortality in Those with ≥2 Years’ Renal Functional Follow-Up Data According to Baseline HbA_1c_, uACR and Plasma sTNFR1 after Adjustment for Conventional Risk Factors for Renal Functional Decline in the Study Cohort (n=87).^a^**

| **Variables** | **Cox proportional hazards regression** | | | | | | |
| --- | --- | --- | --- | --- | --- | --- | --- |
|  | **Clinical model^b^** | | | **Clinical + sTNFR1 model^c^** | | | **Likelihood ratio p-value^d^** |
|  | **HR** | **95% CI** | **p** | **HR** | **95% CI** | **p** |  |
| **≥40% decrease in CKD-EPI eGFR** |  | | | | | | 0.33 |
| HbA_1c_ | 1.04 | 1.00-1.08 | 0.07 | 1.04 | 1.00-1.08 | 0.07 |  |
| uACR | 1.56 | 1.19-2.05 | **0.001** | 1.55 | 1.18-2.03 | **0.002** |  |
| sTNFR1 | N/A | N/A | N/A | 0.64 | 0.26-1.56 | 0.33 |  |
| **Doubling of serum creatinine** |  | | | | | | 0.52 |
| HbA_1c_ | 1.02 | 0.96-1.08 | 0.57 | 1.01 | 0.96-1.08 | 0.64 |  |
| uACR | 1.90 | 1.26-2.87 | **0.002** | 1.87 | 1.24-2.84 | **0.003** |  |
| sTNFR1 | N/A | N/A | N/A | 0.67 | 0.20-2.25 | 0.51 |  |
|  | **Logistic regression** | | | | | | |
|  | **Clinical model^b^** | | | **Clinical + sTNFR1 model^c^** | | |  |
|  | **OR** | **95% CI** | **p** | **OR** | **95% CI** | **p** |  |
| **≥40% decrease in CKD-EPI eGFR** |  |  |  |  |  |  | 0.29 |
| HbA_1c_ | 1.02 | 0.97-1.07 | 0.39 | 1.02 | 0.98-1.08 | 0.34 |  |
| uACR | 1.64 | 1.18-2.39 | **0.005** | 1.66 | 1.18-2.44 | **0.006** |  |
| sTNFR1 | N/A | N/A | N/A | 0.49 | 0.12-1.81 | 0.29 |  |
| **Doubling of serum creatinine** |  |  |  |  |  |  | 0.35 |
| HbA_1c_ | 1.01 | 0.94-1.07 | 0.86 | 1.00 | 0.94-1.07 | 0.89 |  |
| uACR | 2.09 | 1.31-3.69 | **0.004** | 2.08 | 1.30-3.69 | **0.005** |  |
| sTNFR1 | N/A | N/A | N/A | 0.46 | 0.08-2.30 | 0.35 |  |

^a^95% CI = 95% confidence interval; CKD-EPI = Chronic Kidney Disease-Epidemiology Collaboration; eGFR = estimated glomerular filtration rate; HbA_1c_ = glycated haemoglobin; HR = hazard ratio; N/A = not applicable; OR = odds ratio; sTNFR1 = soluble tumour necrosis factor receptor-1; uACR = urine albumin-to-creatinine ratio.

^b^Clinical model: age, gender, diabetes duration, systolic blood pressure, HbA_1c_, CKD-EPI eGFR, uACR.

^c^Clinical + sTNFR1 model: clinical model + plasma sTNFR1.

^d^Clinical model versus clinical + sTNFR1 model.

^e^Composite endpoint 1: ≥40% decrease in CKD-EPI eGFR, doubling of serum creatinine, renal replacement therapy, or mortality.

^f^Composite endpoint 2: doubling of serum creatinine, renal replacement therapy, or mortality.
